# The Concept of a 3D-Printed Microfluidic Device on Oxyfluorinated PDMS Substrates

**DOI:** 10.3390/polym17223044

**Published:** 2025-11-17

**Authors:** Fedor Doronin, Georgy Rytikov, Andrey Evdokimov, Mikhail Savel’ev, Anna Rudakova, Yuriy Rudyak, Victor Nazarov

**Affiliations:** Faculty of Printing Industry, Moscow Polytechnic University, 107023 Moscow, Russia

**Keywords:** composite filament, FFF, microfluidics, oxyfluorination, adhesion, PETG, PDMS

## Abstract

We present the concept of a microfluidic device manufactured using 3D printing and oxyfluorination techniques. During prototype testing, it was found that a larger number of cells adhered to the oxyfluorinated surface compared to the original one. It has also been shown that longer gas-phase treatments correspond to a higher level of cell growth. These items can be used in experiments with reagents and/or microorganisms that cause glass surface corrosion. This increases the number of production techniques for microfluidics devices, expands the possibilities for their use in biotechnology, and solves the main problem of low interlayer adhesion between components of polymer-made microfluidic devices.

## 1. Introduction

An urgent task of modern biotechnology is the controlled cultivation of various cell cultures [1,2,3]. This makes it possible to comprehensively study pathogenic microorganisms, create specific drugs, and obtain specific proteins [4,5,6,7]. As a rule, biotechnological experiments are conducted under well-controlled conditions (temperature, nutrient composition, duration of colony development, etc.) in specialized bioreactors [8,9,10]. Miniaturized bioreactor sizes allow fluctuations in the values of biotechnical parameters to reduce and the level of safety to increase with regard to risks of accidental exposure to pathogens for researchers [11,12,13]. Due to their small size, microfluidic devices are easier to disinfect and dispose of if necessary.

In modern biotechnology, the production of microfluidic bioreactors faces a set of interrelated limitations that significantly affect the effectiveness of their practical application. The key challenge is the problem of scaling up production during the transition from laboratory prototypes to industrial production. Maintaining the required characteristics and ensuring the reproducibility of parameters with increasing production volumes remains a difficult technical task, which directly affects the final cost of products.

Based on photolithography and soft lithography, traditional methods of microfluidic device manufacturing are characterized by significant time and financial costs. It significantly limits the possibilities of production scaling and complicates the integration of various functional elements into a single system. In particular, creating reliable connections between microchannels and external ports as well as integrating sensor elements and monitoring systems is a non-trivial technical task. A promising area of modern applied materials science is the development of microfluidics based on flexible and/or deformable polymer materials [14,15,16].

The typical materials for creating prototypes include polyethylene terephthalate (PET), polyethylene (PE), polypropylene (PP), and polyvinylchloride (PVC) as the substrates, with acrylonitrile-butadiene-styrene copolymer (ABS), polylactide (PLA), polyethylene terephthalate glycol (PETG), and thermoplastic polyurethane (TPU) as the filaments [17,18,19,20,21,22,23,24]. The classical methods of regulating these materials’ structures and properties are plasma chemical treatments [25,26,27] and gas-phase oxyfluorination [28,29,30].

The polymer matrices have suitable gas permeability and high mechanical strength, chemical stability, and biostability when operating under conditions close to normal [31,32,33,34,35,36]. And they can also be subjected to various modifications that provide specific functional properties that enhance the effectiveness of a particular biotechnology [37,38,39,40,41,42]. Another important property of polymer materials is the ability to control wetting with various liquids and the adhesion of components of colloidal solutions (inorganic and/or organic ingredients, agglomerates of biological and non-biological origin) to corresponding surfaces [43,44,45,46,47,48]. The number of structural elements of the colloidal solution adhering to the surface of the bioreactor chamber is an indicator of the practical significance of the polymer material for manufacturing medical and/or biological products [49,50,51]. In most cases, this parameter is evaluated by researchers on a point scale in accordance with the developed technological regulations and/or industry standards [52,53,54]. We propose using the original techniques of image analysis [55,56] to compare the effectiveness of primary surface colonization and surface colonization with oxyfluorinated polydimethylsiloxane (PDMS) by EA.hy926 cell culture. The use of PDMS as a substrate for growing cell cultures is due to the well-known biocompatibility of this polymer [57,58,59].

Existing microfluidic materials often exhibit insufficient biocompatibility and can interact with biological samples, distorting research results. The developed technology partially solves this problem by using polymer materials (ABS, PLA, TPU, PETG), which undergo special gas-phase surface modification. This improves the adhesion between the layers of the structure increases the biocompatibility of the surfaces in contact with cells.

The operational limitations of traditional microfluidic systems are manifested in a limited service life, difficulties with cleaning and sterilization without damaging microstructures, as well as in maintaining stable environmental parameters. The developed device demonstrates the improved surface wettability, increased adhesion of cell cultures, and preservation of mechanical strength after modification.

The use of 3D printing makes it possible to create devices with complex geometry and high precision of execution, while providing the possibility of rapid prototyping and significantly reducing costs in the production of small batches. The economic efficiency of the proposed method is due to minimizing the material consumption, simplifying production scaling, and reducing development time for new structures. This makes the technology accessible to research groups and industrial enterprises.

The biological aspects of our technology include both the effective cell attachment to the bioreactor surface and the real-time monitoring of cell growth possibility. This creates optimal conditions for cell culture development and research in cell engineering.

The purpose of our work was to create, manufacture, and study a prototype of microfluidic bioreactor technology that would be used for the controlled cultivation of the EA.hy926 cell line.

## 2. Materials and Methods

The main stages of the technology [60,61,62] of the microfluidic bioreactor additive manufacturing are as follows: the microfluidic device digital design; the rational choice of the substrate, filament, ingredients, and adhesives materials and the oxyfluorination and/or plasmochemical surface treatment of the substrate; the bulk modification of the filaments and other technological operations; and the microfluidic bioreactor prototype 3D-printing.

### 2.1. The Digital Design and Additive Manufacturing of Microfluidic Bioreactor

**Figure 1:**
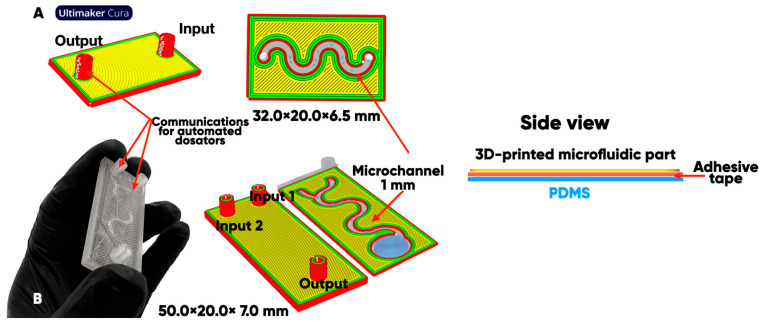
The configuration of microfluidic devices on a polydimethylsiloxane (PDMS) substrate with a two- (**A**) or three-component feeding system (**B**) [56].

It is known [63,64,65] that flexible polymer-based microfluidics devices usually consist of several layers that differ in chemical, geometric, and functional properties. The microfluidic chip was designed on the surface of oxyfluorinated polydimethylsiloxane (PDMS) with a two- (Figure 1A) or three-component feeding system (Figure 1B). It is a single-channel module with a 1 mm-wide zigzag channel that changes its color indication at temperatures above 37 °C [66]. This is necessary to solve several applied problems in microfluidic systems that require gradient temperature control within the microchannel.

The final PDMS choice as a substrate material was due to its well-known biocompatibility [67,68,69], accompanied, however, by high hydrophobicity preventing the formation of multilayer products. The plasma chemical treatment and the oxyfluorination technologies [29,30,70,71,72,73] have been applied to enhance the adhesion of polymer materials’ made components to each other [30,74,75,76] and the wetting capacity of the polymer films’ surface with water-containing liquids [27,77,78,79].

**Figure 2:**
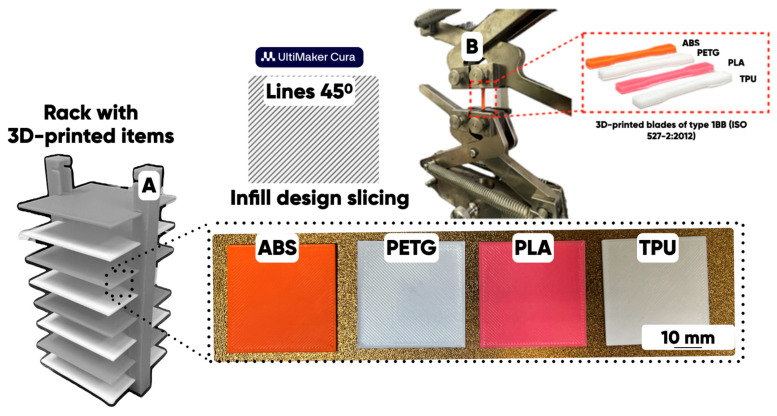
The configuration of 3D-printed items (40 × 40 × 0.8 mm) (**A**) and 3D-printed blades of type 1BB (**B**) [56].

A series of experimental 3D-printed microfluidic chips, items, and blades (Figure 2) were manufactured from initial and composite-containing thermochromics microcapsule [66] filaments by the Anycubic Kobra Go FFF-3D printer (Anycubic, Shenzhen, China), at a temperature of 220–250 °C and a printing speed of 20–40 mm/s.

A copolymer of acrylonitrile, butadiene, and styrene (ABS), and polyethylene terephthalate glycol (PETG), polylactide (PLA), and thermoplastic polyurethane (TPU) were studied as the alternatives for a microfluidic bioreactor additive manufacturing solution supply system (Table 1).

### 2.2. Direct Oxyfluorination of 3D-Printed Items

**Figure 3:**
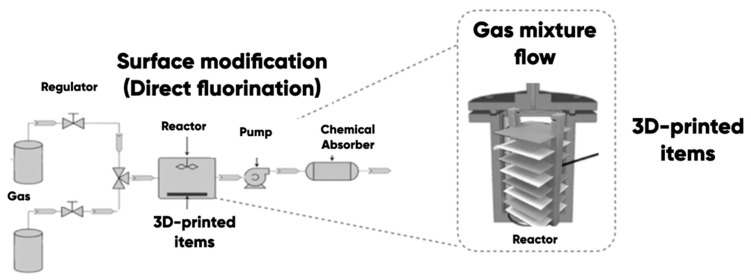
The scheme of 3D-printed items and polymer substrate with direct oxyfluorination.

The modification of the 3D-printed ABS-, PLA-, TPU-, PETG-items, and PDMS-, LDPE-, and others polymer substrates [29] was carried out using direct oxyfluorination (Figure 3). A gas mixture of 7.5 vol.% of fluorine, 82.5 vol.% of helium, and 10 vol.% of oxygen was used in accordance with the procedure [80] for the sample surface modification. The gas-phase durations were 15, 30, and 60 min.

The samples’ surface was wiped with the ethyl alcohol and dried under normal conditions for a day. Then, the experimental samples were placed in a reaction chamber and degassed by vacuuming. A gas mixture with a specific reagent volume ratio was supplied into the reactor. The gas-phase reaction products were removed from the chamber after the planned time interval and were disposed of using standard chemical absorbers.

### 2.3. Investigation of the 3D-Printed Items and Microfluidic Devices Properties

The changes in the chemical structure of the 3D-printed items and microfluidic device components due to the direct oxyfluorination was studied using a Simex FT-801 IR spectrometer (Novosibirsk, Russia). The polymer substrate edge angles with the distilled water and the ethylene glycol were measured using the specialized KSVCAM 101 equipment (KSV Instruments, Helsinki, Finland). The gas-phase treatment effect on the 1BB-type [81,82] 3D-printed blades’ deformation strength was evaluated using a Zwick Roell BZ1.0 universal bursting testing machine (ZwickRoell Group, Kennesaw, GA, USA). The straining friction coefficient was measured using a universal friction machine (MTU-01, (JSC Concern Nanoindustria, Saint Petersburg, Russia)).

We investigated the interactions between the technology of polymer substrates under oxyfluorination and the adhesion of EA.hy926 cells (ATCC, CRL-2922) to the device surface. The cultivation was carried out in DMEM medium supplemented with 4.5 g/L glucose, 10% inactivated fetal bovine serum, 50 μg/mL gentamicin, 2 mmol/L L-glutamine, 1% NEAA, and the growth factor HAT. The cultivation took place at 37 °C in a 5% CO_2_/95% air atmosphere with subculture twice per week. The cell adhesion on the 3D-printed microfluidic channel was monitored using a Stemi 2000 stereomicroscope (Carl Zeiss Microscopy GmbH, Munich, Germany). The experimental samples’ surface wetting edge angles with the distilled water and the ethylene glycol were measured using the specialized KSVCAM-101 equipment. The specific surface free energy $\gamma_{S}\left(mJ/m^{2}\right)$ calculation (its polar $\gamma_{S}^{P}$ and disperse $\gamma_{S}^{D}$ components) was carried out using the Owens–Wendt–Rabel–Kaelble (OWRK) technique taking into account the Girifalco–Good–Fowkes model.

## 3. Results and Discussion

### 3.1. Experimental Samples Characterization

**Figure 4:**
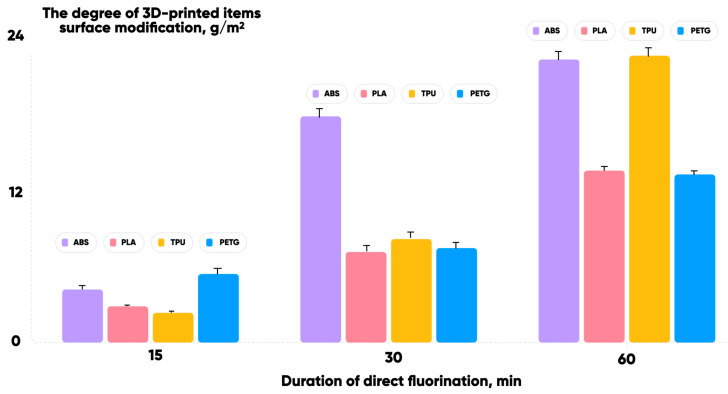
The degrees of the surface modification $C_{G}^{M}\left(g/m^{2}\right)$ obtained at different times of 3D-printed items.

The results of ABS/PLA/TPU/PETG 3D-printed items under direct oxyfluorination for 15, 30, and 60 min are presented in Figure 4.

**Figure 5:**
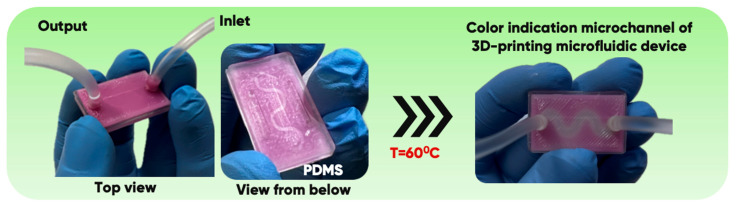
PETG-composite microfluidic chip with a color-indicating thermochromic microchannel on an oxyfluorinated PDMS substrate. The color change in the microchannel is due to the flow of functional fluids at a temperature of 37 °C.

The gas-phase modification was carried out under the same conditions for a set of 10 samples of each polymer fixed in a tool container. The degree of modification $C_{G}^{M}\left(g/m^{2}\right)$ was determined as the ratio of gas-phase treatment-induced sample mass increment to its total surface area. It increased for all the experimental samples. And the corresponding water and ethylene glycol contact angles decreased [76]. This provides an opportunity to create microfluidic reactors of different sizes and shapes using FFF-based additive manufacturing technology (Figure 5).

**Figure 6:**
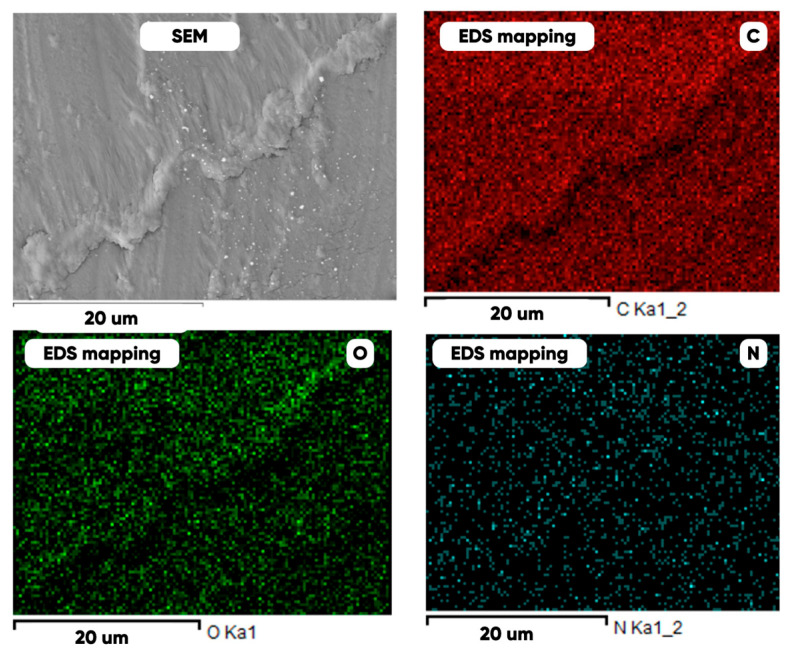
SEM analysis and EDS-mapping PETG filament with thermochromic microcapsules [66].

As can be seen in Figure 5, the created microfluidic chip has an amazing ability to display the colors. It is able to transport liquids with temperatures above 37 °C which makes it particularly useful for applications in various fields. This impressive result has been achieved thanks to the use of PETG filaments we have previously developed and the uniform distribution of thermochromic microcapsules throughout the polymer matrix of the filament (Figure 6). SEM analysis and EDS-mapping data have confirmed that the microcapsules are distributed very evenly [66].

The high degree of uniformity in the distribution of microcapsules within the polymer allows us to directly control the color characteristics of the microfluidic chip. This opens up new possibilities for further development and applications. It can be seen that with a short modification time the PETG oxyfluorination is the most effective and the TPU is the least effective (Figure 4). However, with a longer treatment duration the larger changes in the samples’ surface modification degree are typical for TPU and ABS, and the smaller ones for PLA and PETG. This means that there is a significant change in the chemical and physical structure of the studied 3D-printed materials’ surface layers and polymer substrate (Table 2) during gas-phase processing.

**Figure 7:**
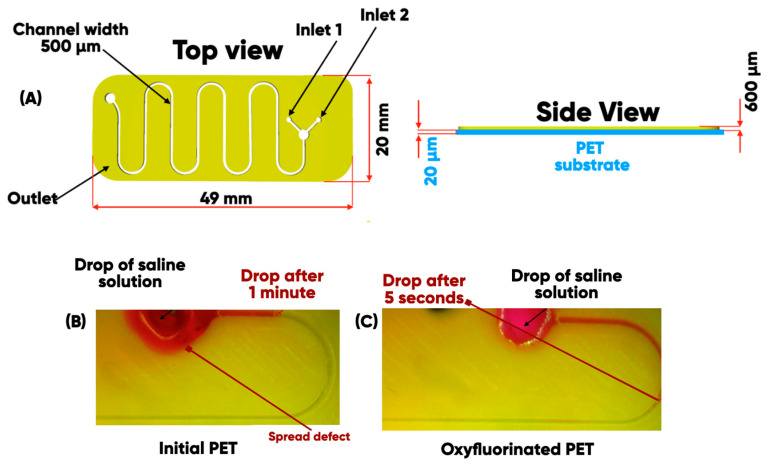
3D-printed planar microfluidic device (**A**) and the process of spreading drops of saline solution onto on initial (**B**) and oxyfluorinated (**C**) PET substrates [76].

It can be seen [76] that PET-oxyfluorination is the most effective for both a short and long duration of the gas-phase treatment for the developed 3D-printed microfluidic device on polymer substates (Figure 7A).

The experimental results demonstrate significantly different behaviors of a saline solution on two distinct types of surfaces. On an initial PET substrate (Figure 7B), there is no diffusion of the liquid through microchannels, and the droplet retains its original shape. This phenomenon is attributed to the low surface energy of the substrate and its high wetting angle. Conversely, on a modified oxyfluorinated PET substrate (Figure 7C), the solution spreads more readily and extensively through the microchannels. This process is most active in the initial 30 s following contact, after which it significantly decreases due to the limited volume of supplied saline solution.

**Figure 8:**
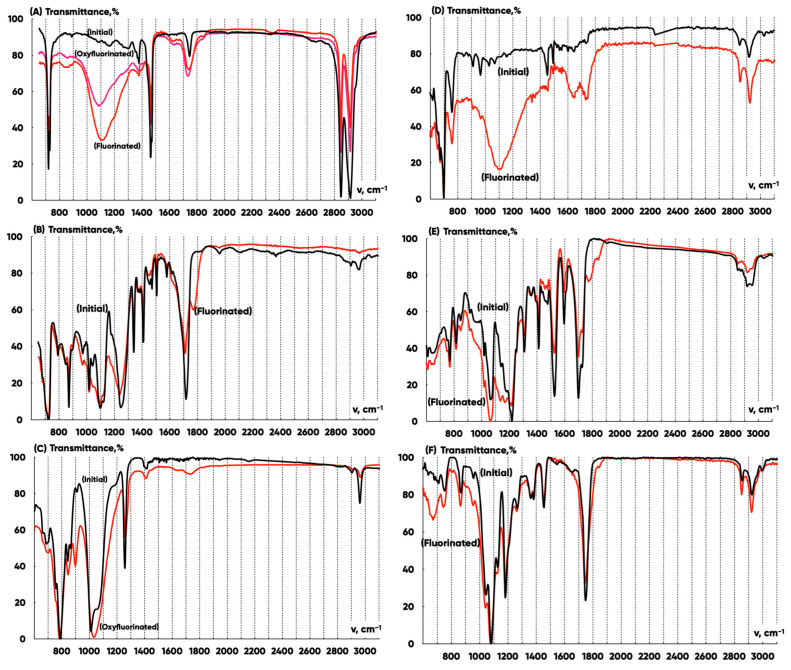
IR spectra of initial, fluorinated, and oxyfluorinated LDPE (**A**), PETG (**B**), and PDMS (**C**) substrates and ABS (**D**), TPU (**E**), and PLA (**F**) 3D-printed items.

The comparative analysis of the infrared radiation transmittance spectra (Figure 8) for the LDPE, PET, and PDMS substrates and ABS, PLA, and TPU 3D-printed items allowed us to establish that the modification with fluorine and oxygen-containing gas mixtures in technologically accessible ranges of reagent concentration and treatment duration significantly changes the chemical structure of the LDPE, PDMS, and ABS but has little effect on the chemical structure of PET, PETG, and TPU. This is probably due to the difference in the relative proportion of unsaturated and CH-bonds of the molecular energy structure, the diffusion coefficients, and the permeability of the reagents into the polymers’ matrix.

The effective absorption bands of IR radiation with wave numbers ~3030–3050 ${cm}^{-1}$ and ~2950–3000 ${cm}^{-1}$ characterize the valent vibrations of σ-bonds in $\left(–CH_{3}\right)$ and $\left(–CH_{2}–\right)$ groups of macromolecules. The deformation vibrations of CH–bonds are indicated by a group of lines in the ~750–790 ${cm}^{-1}$ range. As a result of the terminal groups’ hydrogen atoms’ substitution with the fluorine ones, the absorption intensity decreases in the range of ~3030–3050 ${cm}^{-1}$, and the $\left(–H_{2}CF\right)$ groups’ hydrogen atoms valent vibrations lines ~2940, ~2935 and CF– bonds valent vibrations lines ~1025, ~1075 appear in the spectrum. These latter lines transform into a wide band of ~800–1300 ${cm}^{-1}$ when the degree of modification increases. The carbonyl groups’ π-bonds valent vibrations can be identified by the ~1786 (±6) ${cm}^{-1}$ line. The hydroxyl groups’ formation is reflected by the ~1050 ${cm}^{-1}$ and ~1150 ${cm}^{-1}$ lines. The carboxylic groups’ vibrations correspond to a line with the ~685 ${cm}^{-1}$ wave number.

Thus, during the $He/F_{2}/O_{2}=82.5/7.5/10\;vol.\%$ gas mixture treatment, the following takes place:

- Fluorine atoms replace hydrogen ones effectively in $\left(–CH_{3}\right)$ and $\left(–CH_{2}–\right)$ macromolecules’ groups. The absorption decreases in ~2950–3050 ${cm}^{-1}$ and grows in the ~800–1300 ${cm}^{-1}$ wave number ranges for all the samples;
- (–C=O) groups are formed. The absorption increases for all samples in the ~1750–1810 ${cm}^{-1}$ wave number range;
- $\left(–CH(OH)–\right)$ and $\left(–CH_{2}(OH)\right)$ groups appear. The absorption increases for all the samples in the ~1000–1200 ${cm}^{-1}$ wave number range.

The analysis of the elemental composition confirms the fundamental restructuring of the PETG-composite surface layer [83] as a result of fluorination (Table 3).

This is expressed in a significant decrease in the mass fractions of carbon (from 76.1% to 64.7%) and oxygen (from 23.2% to 21.6%), with the simultaneous appearance of fluorine at a content of 13.0%.

This redistribution of elements clearly indicates the successful integration of fluorine atoms into the polymer matrix. This leads to a radical change in the properties of the polymer substrate.

The specific free surface energy of the contacting microfluidic bioreactor components increased due to the gas-phase modification and caused chemomorphological transformations. It provides an opportunity to achieve the adhesive strength sufficient to form a prototype of the product. But it should be noted that the carboxyl groups are present in both the initial and the modified polymers. And it indicates some natural oxidation of polymer materials during the storage and a slight change in the element’s composition proportion under the gas-phase modification.

### 3.2. Oxyfluorination Effect on the 3D-Printed Samples’ Properties

The changes of friction coefficient $K_{f}$ and wetting with different polarity liquids due to the gas-phase modification are statistically significant for the 3D-printed items made from ABS, PLA, TPU, and PETG (Table 4).

The effect of a fluorine and oxygen-containing gas mixture on the surface roughness of the experimental samples is multidirectional: the main roughness parameter $R_{a},nm$ increases for ABS, decreases for PETG, and practically does not change for TPU and PLA, taking into account the measurements’ statistical error. Consequently, the significant changes in the surface frictional and adhesive properties are caused by an increase in specific free energy value. And it is caused not so much by a change in the average roughness but by the modification-induced surface textural mode and chemical structure transformations.

A number of studies [84,85,86,87] have shown that fluorination of polymer materials under suboptimal conditions can lead to loss of mechanical strength from the products. The direct measurement technique (GOST 11262-2017) shows (Table 5) that the surface oxyfluorination under the experimental conditions does not affect the tensile strength of the 3D-printed blades made from ABS, PLA, TPU, and PETG filaments (the corresponding values remain unchanged within the statistical error).

The improvement in function and the preservation of structural characteristics makes it possible to use a combination of additive prototyping and gas-phase treatment techniques for the manufacture of multilayer microfluidic products. And we have shown it with the example of a 3D-printed microfluidic device concept.

**Figure 9:**
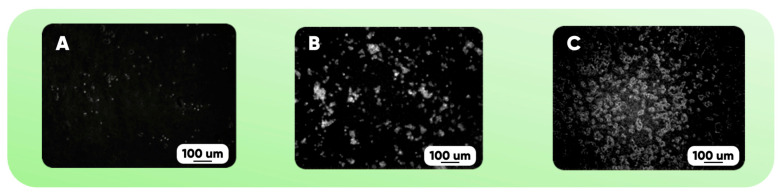
The dependence of the EA.hy926 cell culture development on the duration of the microfluidic bioreactor PDMS-substrate oxyfluorination: on the initial sample (**A**); after modification for 15 (**B**) and 60 (**C**) minutes with He/F_2_/O_2_ = 82.5/7.5/10 vol.% gas mixture.

The results of the microfluidic bioreactor prototype functionality testing are shown in Figure 9. High-contrast negative photographing of the surface of the PDMS-substrate made it possible to establish that a greater duration of oxyfluorination corresponds to a greater number of cells adhering to the surface of the manufactured device.

The following technique was used to calculate the number of observed cells:

(1) For type A images, in which cells can still be seen separately, the number of pixels representing one cell was determined (values between 40 and 70 pixels were obtained).
(2) A representative image region was chosen for which the average pixel brightness and its standard deviation were calculated.
(3) To estimate the number of observed cells, the total number of pixels whose brightness exceeds the sum of the average value and standard deviation was divided by the number of pixels used to display one cell.
(4) Paragraphs 2 and 3 were repeated for the following representative image fragments in the working cycle of the computing script.

The characteristic cell sizes ranged from approximately 10 to 20 microns. The number of cells observed ranged from 45 to 60 (Figure 9A), 120 to 160 (Figure 9B), and 180 to 240 (Figure 9C) on the representative fragments of Figure 9 images.

It can be seen that the number of cells adhering to the oxyfluorinated surface is much higher than to the original one. Moreover, a longer duration of the gas-phase modification corresponds to a larger number of cells. The observed differences in the forms of bioagglomerates indicate a significant effect of the chemical structure of the oxyfluorinated surface on the possibility of the effective cell culture cultivation (Table 6).

Analysis of the data in Table 5 indicates that the duration of oxyfluorination directly affects the wettability and surface energy of PDMS. When the treatment time is increased to 60 min, there is a noticeable improvement in wettability, as evidenced by a consistent decrease in wetting angles for both water and ethylene glycol. This improvement is linked to an increase in the total specific free surface energy from 25.7 mJ/m^2^ to 34.9 mJ/m^2^.

The most significant change is a dramatic increase in the polar energy component from 1.9 mJ/m^2^ to 10.8 mJ/m^2^, while the dispersion component remains relatively constant. This suggests that oxyfluorination enhances the hydrophilicity of PDMS surfaces by introducing polar functional groups. These modifications in properties are most significant at a treatment duration of 60 min.

**Figure 10:**
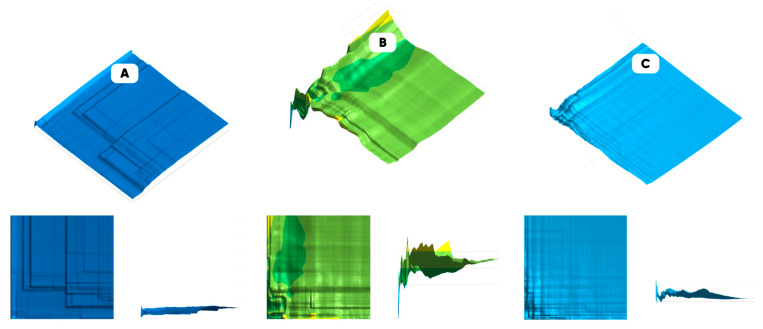
The images’ variational representativeness maps for the results of EA.hy926 cell culture growing at different times with the oxyfluorinated PDMS substrate of the 3D-printed microfluidic device: the initial (**A**) and the He/F_2_/O_2_ = 82.5/7.5/10 vol.% gas mixture modification for 15 (**B**) and 60 (**C**) minutes.

The representativeness of the data was assessed based on the obtained optical images and computer analysis. The characteristic size of the cultured cells’ planar distribution morphological heterogeneity was determined. And the morphological spectra of the surface of the experimental samples were constructed. Figure 10 shows the maps of the representativeness for the cell culture samples’ populated surfaces.

It can be seen that the largest value of the deposited cells along the vertical axis pixel lightness variation coefficients refers to the sample with the higher number of individual cells and/or cellular agglomerates. The frontal and the profile projections of the constructed “variational representativeness maps” are presented in the corresponding insets.

**Figure 11:**
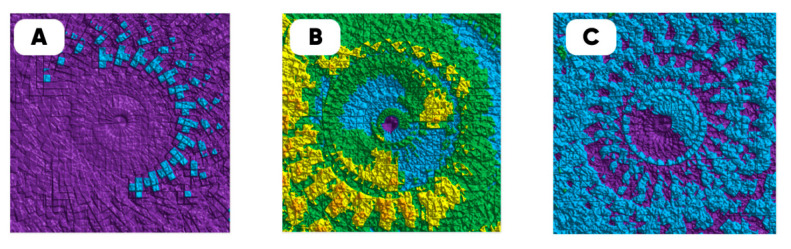
The variation-rotational pictures characterizing the optical uniformity degree of the samples’ surface (on which EA.hy926 cell culture developed under the same conditions): initial (**A**) and oxyfluorinated He/F_2_/O_2_ = 82.5/7.5/10 vol.% for 15 (**B**) and 60 (**C**) minutes.

Figure 11 shows the variation-rotational patterns (VRP) visualizing the results of calculating the pixel brightness variation coefficients for the experimental samples’ image clones, formed as a result of flat rotation at angles from 0 to 180 in increments of 15 degrees [88]. The violet color indicates the set of pixels in the range for which the variation coefficient does not exceed the 0.5 value.

A smaller area of optical uniformity (indicated in purple) refers to the VRP of populated with the cell culture 15 min oxyfluorinated PDMS substrate (Figure 11B).

**Figure 12:**
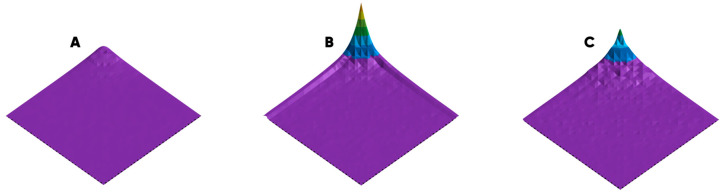
The morphological spectra characterizing the populated surface with EA.hy926 cell culture sample surface’s heterogeneity mode structure: initial (**A**) and oxyfluorinated He/F_2_/O_2_ = 82.5/7.5/10 vol.% for 15 (**B**) and 60 (**C**) minutes. The violet color indicates a set of biharmonic amplitudes that form morphological noise that is not distinguishable to the naked eye.

Figure 12 shows the averaged morphological spectra [89] of the populated surface by EA.hy926 experimental sample’s surface.

The large values of the morphological spectrum biharmonic average amplitudes correspond to a more developed heterogeneity mode structure of the biomodified surface. A summary of the quantitative characteristics of the EA.hy926 cell culture sample surface colonization degree is presented in Table 7.

The greatest morphological heterogeneity refers to the samples subjected to gas-phase treatment with the fluorine- and the oxygen-containing gas mixtures for 15 min. However, the approximation of the variation coefficient on the treatment modification duration and empirical dependence with the quadratic and Gaussian models indicates the highest probability of achieving the maximum heterogeneity value with ~30 (±2) minutes of gas-phase processing time.

## 4. Conclusions

A PETG-composite microfluidic chip with a color-indicating thermochromic microchannel on an oxygen-fluorinated PDMS substrate has been developed. All microfluidic bioreactor prototype components have been subjected to surface modification, which provides increased adhesion between the layers of the 3D product. The treatment has been performed using a fluorine- and oxygen-containing gas mixture. The effectiveness of the modification has been evaluated gravimetrically and by IR spectroscopy.

The comparative analysis of the IR transmission spectra of LDPE-, PET-, and PDMS-substrate samples and 3D-printed ABS-, PLA-, TPU-, and PETG-test products allowed us to determine that modification with fluorine- and oxygen-containing gas mixtures significantly alters the chemical structure of LDPE, PP, and PDMS. However, it has a minor effect on the chemical structure of PETG, PLA, and TPU, which is likely due to the relative proportion of unsaturated bonds and CH-groups in the molecular structure of these polymers.

The direct measurements of the physical and mechanical properties of the modified ABS-, PLA-, TPU-, and PETG-test products allowed us to establish that treatment with fluorine- and oxygen-containing gas mixtures does not affect tensile strength but leads to statistically significant changes in friction coefficient and wettability of surfaces with different polarity liquids.

Microfluidic bioreactor prototypes were tested using EA.hy926 cells as an example of cell culture growth. It was found that a higher number of cells adhere to the oxyfluorinated surface compared to the original surface. Additionally, a longer gas-phase treatment corresponds to a higher level of cell culture development.

In the future, we plan to optimize the production technology of similar devices by optimizing the composition of gas mixtures and the duration of treatment.

## Figures and Tables

**Table 1:** Material extrusion technological parameter of the additive manufacturing process.

Filaments	Manufacturer	Technological Parameter of FFF 3D-Printing		
		Temperature of Extrusion, °C	Build Platform Temperature, °C	Printing Speed, mm/min
Acrylonitrile, butadiene, and styrene copolymer (ABS)	Shenzhen Esun Industrial Co., Shenzhen China	250	80	40
Polylactide (PLA)		210	50	40
Polyethylene terephthalate glycol (PETG)		235	60	40
Thermoplastic polyurethane (TPU)	U3print, Moscow, Russia	210	60	30
Polyethylene terephthalate glycol (PETG) containing 1 mass.% of thermochromic microcapsules	Moscow Polytechnic University, Moscow, Russia [66]	240	60	40

**Table 2:** The degrees of the modification $C_{G}^{M}\left(g/m^{2}\right)$ obtained at different times of LDPE/PP/PVC/PET substrate treatment with direct oxyfluorination.

	$\mathrm{The}\;\mathrm{Degree}\;\mathrm{of}\;\mathrm{Polymer}\;\mathrm{Substrates}’\;\mathrm{Surface}\;\mathrm{Modification},\;g/m^{2}$			
Duration of Oxyfluorination, min	PET	PP	PVC	LDPE
30	0.40 ± 0.07	0.36 ± 0.04	0.19 ± 0.03	0.23 ± 0.03
60	1.3 ± 0.2	0.63 ± 0.07	0.37 ± 0.05	0.35 ± 0.04
90	1.4 ± 0.2	0.69 ± 0.08	0.46 ± 0.06	0.43 ± 0.04
180	1.6 ± 0.4	0.73 ± 0.08	0.48 ± 0.06	0.49 ± 0.06

**Table 3:** EDS analysis and distribution of chemical elements on the surface of initial and fluorinated PETG with shungite [83].

Elemental Composition, mass. %				
C	O	F	Al	Si
**Initial**				
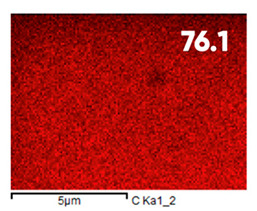	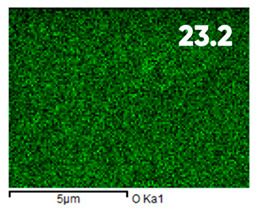	-	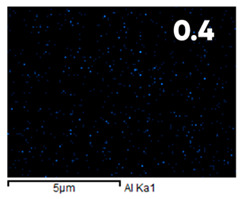	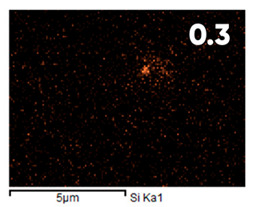
**Fluorinated**				
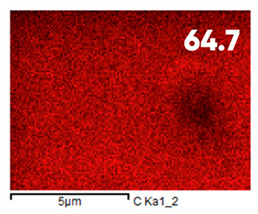	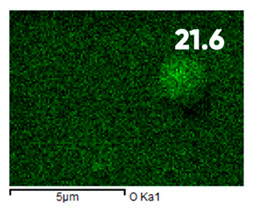	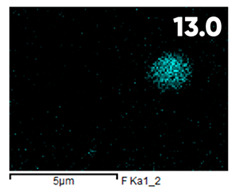	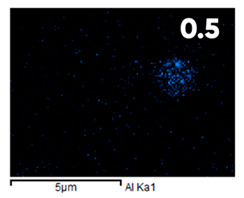	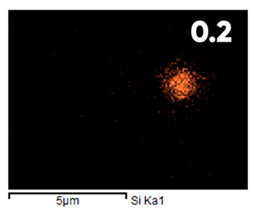

**Table 4:** The water ${\theta_{w}}^{o}$ and ethylene glycol ${\theta_{e}}^{o}$ wetting edge angles, the friction coefficient $K_{f}$, and the average roughness $R_{a},nm$ of the 3D-printed ABS-, PLA-, TPU-, and PETG-items’ surfaces before and after direct oxyfluorination.

3D-Printed Items	ABS	PLA	TPU	PETG
Parameter	Initial/Oxyfluorinated			
${\theta_{w}\pm \sigma_{\theta_{w}},}^{o}$	67 ± 7/45 ± 5	74 ± 7/52 ± 5	85 ± 9/48 ± 5	79 ± 8/43 ± 4
${\theta_{e}\pm \sigma_{\theta_{e}},}^{o}$	62 ± 6/18 ± 2	46 ± 5/23 ± 2	48 ± 5/20 ± 2	51 ± 5/17 ± 2
$R_{a}\pm \sigma_{R_{a}},nm\;$	6.8 ± 0.7/9.8 ± 0.9	5.9 ± 0.6/4.9 ± 0.5	4.9 ± 0.5/4.6 ± 0.5	12 ± 1/8.0 ± 0.8
$K_{f}\pm \sigma_{K_{f}}$	1.2 ± 0.1/0.22 ± 0.02	0.5 ± 0.05/0.21 ± 0.02	0.85 ± 0.08/0.41 ± 0.04	1.1 ± 0.1/0.43 ± 0.04

**Table 5:** The tensile strength (MPa) of 1BB-type 3D-printed blades from TPU, ABS, PETG, and PLA after direct oxyfluorination.

3D-Printed 1BB Blades	Duration of Direct Oxyfluorination, min									
	0	5	10	15	20	25	30	45	60	90
TPU	36 ± 4	38 ± 4	37 ± 4	39 ± 5	40 ± 4	41 ± 4	40 ± 4	40 ± 5	41 ± 4	40 ± 5
ABS	41 ± 4	43 ± 5	42 ± 4	44 ± 5	43 ± 6	43 ± 6	41 ± 4	42 ± 6	43 ± 4	42 ± 5
PETG	52 ± 5	51 ± 6	53 ± 5	52 ± 5	53 ± 5	51 ± 6	52 ± 5	52 ± 5	52 ± 6	51 ± 6
PLA	64 ± 6	66 ± 7	67 ± 5	68 ± 7	67 ± 6	68 ± 7	67 ± 5	67 ± 6	65 ± 7	66 ± 7

**Table 6:** The water ${\theta_{w}}^{o}$ and ethylene glycol ${\theta_{e}}^{o}$ wetting edge angles and the corresponding values of the specific free surface energy (total, dispersion, and polar components) for initial and oxyfluorinated PDMS-substrates.

Material	Oxyfluorination Duration (minutes)	Wetting Edge Angle, °		$\gamma ,mJ/m^{2}$	$\gamma_{S}^{D},mJ/m^{2}$	$\gamma_{S}^{P},mJ/m^{2}$
		$\theta_{H_{2}O}$	$\theta_{C_{2}H_{6}O_{2}}$			
PDMS	0	96 ± 9	70 ± 7	25.7 ± 0.8	23.8 ± 0.6	1.9 ± 0.2
	15	94 ± 8	65 ± 7	30.1 ± 0.8	28.5 ± 0.7	1.6 ± 0.1
	30	89 ± 8	60 ± 6	31.6 ± 0.9	29.0 ± 0.7	2.6 ± 0.2
	60	74 ± 7	46 ± 6	34.9 ± 0.9	24.1 ± 0.5	10.8 ± 0.4

**Table 7:** The PDMS substrate morphological heterogeneity quantitative characteristics—initial and oxyfluorinated He/F_2_/O_2_ = 82.5/7.5/10 vol.% for 15 and 60 min with the EA.hy926 cell culture developed.

PDMS	Duration of Direct Oxyfluorination, min		
Parameter	0	15	60
$\bar{L_{ij}}$	20	25	57
$∆\left(L_{ij}\right)$	2	10	35
V	0.23	1.32	0.57
δV	0.15	0.33	0.14
$M\left(A_{kl}\right)$	1.6	19	6.8
$R\left(A_{kl}\right)$	11	5.9	12

Here, $\bar{L_{ij}}$ and $∆\left(L_{ij}\right)$ are the average value and standard deviation of the pixels brightness (Figure 10); V, δV—values of the variation coefficient of variation-rotational patterns (Figure 11); $M\left(A_{kl}\right)$, $R\left(A_{kl}\right)$—the magnitude and the radius of the localization zone of the symmetric Gaussian function used to approximate the morphological spectra (Figure 12).

## Data Availability

Data are contained within the article.
